# Herbal Extract of *Gynostemma Pentaphyllum* Decreases Hepatic Glucose Output in Type 2 Diabetic Goto-Kakizaki Rats

**Published:** 2011-06

**Authors:** K. Yassin, V. T. T. Huyen, K. N. Hoa, C. G. Östenson

**Affiliations:** 1 *Depatment of Molecular Medicine and Surgery, Karolinska Institutet, Stockholm, Sweden;*; 2 *Department of Internal Medicine, Hanoi Medical University, Hanoi, Vietnam;*; 3 *Department of Endocrinology and Diabetes, National Institute of Gerontology, Hanoi, Vietnam;*; 4 *Department of Internal Medicine, University of Manitoba, Winnipeg, Manitoba, Canada*

**Keywords:** glucose tolerance, gynostemma pentaphyllum, herbal medicine, insulin sensitivity

## Abstract

The aim of the study was to explore the effect of *Gynostemma pentaphyllum* (GP) extract on hepatic glucose output (HGO) in spontaneously type 2 diabetic Goto-Kakizaki (GK) rats treated orally with GP or placebo extract 1600 mg/kg daily, during three days or three weeks. The three-week treatment of GP, but not three-day treatment, significantly reduced plasma glucose (PG) levels from 9.8 ± 0.6 to 6.8 ± 0.4 mmol/L (*p*=0.027) in GK rats, whereas PG levels were not significantly decreased in the placebo rats. Glucose tolerance assessed by an intra-peritoneal glucose tolerance test was significantly improved in GP treated compared to placebo treated group (areas under the glucose curves, AUCs, from 0 to 120 min were 1150 ± 200 *vs*. 1761 ± 87 mmol/L; *p*=0.013). The glucose response in an intra-peritoneal pyruvate tolerance test from minute 15 to minute 120, the AUC (15-120) was significantly lower in the GP group (415.5 ± 68.0 *vs.* 641.5 ± 41.8 mmol/L; *p*<0.05). In liver perfusions, the AUCs for HGO during 18 min (0-18 min) were significantly decreased in GP treated rats compared with control rats (302.8 ± 36.5 *vs.* 423.5 ± 44.7 μmol, *p*<0.05). The three-week GP treatment significantly reduced the hepatic glycogen content, but not glycogen synthase activity compared to placebo group (*p*<0.007). In conclusion, three-week treatment of GP extract exerted anti-diabetic effect in GK rats, reducing plasma glucose levels and HGO, suggesting that GP improves the hepatic insulin sensitivity by suppressing gluconeogenesis.

## INTRODUCTION

Type 2 diabetes is a common disease that is characterized by chronic hyperglycemia due to impaired insulin secretion and decreased insulin action (1). Diminished insulin sensitivity in extra-hepatic tissues, such as muscle and adipose tissue, decreases glucose utilization and insulin resistance in liver increases glucose production. Therefore, the liver is an important organ in maintaining glucose homeostasis (2). Thus, hyperglycemia associated with impairments in the regulation of fasting glucose production and glucose tolerance may be improved by therapeutics which target insulin resistance in the liver (3).

Plant extracts have been used in traditional medicine for treatment of various diseases including diabetes (4). *Gynostemma pentaphyllum* (GP) has been used in traditional herbal medicine in Vietnamese and some other Asian countries. GP extract reportedly has numerous effects, such as cholesterol-lowering, immune-potentiating, anti-tumor, anti-oxidant, and anti-diabetic effect (5-8). Our previous studies revealed that phanoside, a gypenoside isolated from the GP, reduced blood glucose in mice and rats (9, 10). Moreover, in a randomized placebo-controlled clinical trial in drug-naïve patients with type 2 diabetes, GP extract significantly improved HbA1c values and fasting plasma glucose levels (11). The latter effect in addition to improvement in insulin sensitivity suggested that the main effect may reside in the liver. To further explore this, we have performed experiments in spontaneously type 2 diabetic Goto-Kakizaki (GK) rats (11-16). The GK rat develops hyperglycemia post-natally and maintains moderately enhanced plasma glucose levels during its lifetime (17). This animal model of type 2 diabetes is characterized by not only major impairment in beta cell function, but also insulin resistance in muscle and liver. This study aims to investigate the direct effect of GP extract on hepatic glucose output, using the perfused GK rat liver.

## MATERIALS AND METHODS

### Animals

Altogether twenty one diabetic Goto-Kakizaki (GK) rats (200-300 g), originating from Wistar rats, were bred in our department. The animals were kept at 22°C on a 12-hour light-dark cycle (6 am and 6 pm) with free access to food and water before being anesthetized for liver perfusion. The study was approved by the Laboratory Animal Ethics Committee of the Karolinska Institutet (N367/08, N72/08).

### Plant material

The whole plants of *Gynostemma pentaphyllum* Makino-Cucurbitaceae were collected from Hoa Binh province, in the north of Vietnam, and identified by Professor Pham Thanh Ky, Department of Material Medica, Hanoi College of Pharmacy. A voucher specimen (HN-0152) was deposited in the herbarium at Department of Material Medica, Hanoi College of Pharmacy.

### Method of extraction

The production of GP extract was processed as specified. Briefly, the process included extraction of the authenticated GP plants for 2h in boiling water, and with a following precipitation of impurities by adding concentrated 70% ethanol. The 70% ethanol was then removed by distillation at low pressure, and impurities were removed by filtration. Thereafter, the extract was inspected as a semi-finished brown powder with typical odour of GP extract. This powder had a humidity of approximately 6.7%, and could be dissolved in water into brown liquid with a sweet-bitter flavour. The extract contained flavonoids, as shown by a positive cyanidin reaction with base FeCl_3_ (5%) and furthermore about 18% saponins, as indicated by a positive foaming test (18, 19). Thus, the standardization of the GP extract included confirmation of its typical odour, state and sweet-bitter flavour, approximately 7% in humidity, and positive reaction in the flavonoid (cyanidin reaction) and saponin (foaming) tests. The placebo was green tea (*Camellia sinensis*), which was supplied at the same dose and was similar to the GP extract in shape and packaging. Both GP and placebo extracts were ground into a soluble powder that easily could be dissolved in water at room temperature.

### Oral administration of GP and placebo extracts

GP and placebo extracts were given to unanesthetized male GK rats by gavage through an enteral feeding tube (polyvinyl chloride, sterile VYCON, Lab. Pharmaceutiques Vycon, Ecouen, France) connected to a syringe with the solutions. The GK rats were divided into two groups in each experiment; 800 mg/kg of GP extract or placebo extract were given twice a day at 9:00 and 15:00 for three days or three weeks. The treatment periods were chosen to reflect short-term and long-term treatment, respectively. The dose was selected from our previous screening study in mice (20) that showed an acute effect of GP with 1500 mg/kg body weight. We thus decided to give GP in a slightly higher total daily dose of 1600 mg/kg, administered as 800 mg/kg two times daily. The two-times-daily dosage was based on our previous experience from a randomized placebo-controlled clinical trial in type 2 diabetic patients (11).

### Plasma glucose (PG) measurement

Blood samples for determination of glucose (about 20 μl/sample) were taken after a small tail incision. Plasma glucose levels were monitored by the glucose dehydrogenase method (Accu-Check Aviva) every 2 days before the morning oral administration of either GP or placebo.

### Intraperitoneal Glucose Tolerance Test (IPGTT) and Intraperitoneal Pyruvate Tolerance Test (IPPTT)

IPGTT and IPPTT were carried out in overnight fasted GK rats. PG concentrations were obtained at 0, 15, 30, 60, and 120 minutes after an intraperitoneal (i.p.) injection of glucose (2 mg/g body weight; Glukos APL 500 mg/ml) or pyruvate (2 mg/g body weight; Sodium pyruvate, SIGMA), respectively.

### Subcutaneous (s.c) insulin tolerance test (SCITT)

For the SCITT, insulin was injected at a dose of 0.5 U/kg and plasma glucose levels were measured in fasted GK rats before the injection of insulin (0 min) and every 15 minutes for 2 hours and then every 30 minutes for another 2 hours.

### Isolation and perfusion of GK rat liver for determination of hepatic glucose output (HGO)

Liver perfusions were started between 10 am and noon. The rats were anesthetized with an i.p. injection of ketamine (60-70 μg/g body weight; Pfizer AB, Täby, Sweden). Livers were perfused *in situ* without recirculation in a 37°C cabinet via the portal vein using Krebs-Henseleit bicarbonate buffer (KRB), pH7.4, which was equilibrated with 95% O_2_ and 5% CO_2_. The perfusion pressure was kept constant with a flow rate of 3.0-4.0 ml/min/g liver.

Adrenaline (Merck AB NM, Stockholm, Sweden) was diluted into the perfusion medium (KRB) to the final concentration of 50 nmol/L. Livers were perfused for 8-18 minutes. In rats treated for three days, livers were perfused with KRB during eight minutes. In rats treated for three weeks, the first eight minute with KRB only was followed by adding adrenalin (50 nmol/L) in KRB for 10 minutes. Samples of the perfusate were taken at 2-minute intervals from the inferior caval vein during perfusion, and their glucose levels were measured by the glucose oxidase method using a glucose analyzer (Yellow Springs Instruments). Hepatic glucose output was calculated using the mean glucose concentration in relation to flow rate and hepatic dry weight. These livers were not used for any other measurements.

### Hepatic glycogen content

Liver homogenates were extracted in 80% ethanol to remove glucose. An aliquot of each homogenate was mixed with amyloglucosidase (Roche Applied Science) and incubated at 60°C for 15 minutes to degrade glycogen into glucose residuals. The samples were diluted and incubated with 1 ml of Glucose Assay Reagent (o-Dianisidine Reagent + Glucose Oxidase/Peroxidase Reagent, Sigma-Aldrich) at 37°C for 30 minutes, followed by the addition of 1 ml 12N H_2_SO_4_ to stop the reaction. The absorbance of glucose was read at 540 nm. In parallel, different concentrations of rabbit liver glycogen type III (Sigma-Aldrich) were treated as the samples and used as standard curve.

### Hepatic glycogen synthase (GS) activity

GS was determined by a method based on incorporation of ^14^C-labelled uridine diphosphate-glucose into glycogen. The active form of GS (that is the form of GS which is activated by insulin) was measured at a low concentration of glucose-6-phosphate (0.3 mm) and the total GS at a high concentration (6.0 mm) (21).

### Statistical analysis

Statistical analyses were carried out with Sigmaplot (2001). The results have been calculated as mean ± SEM and comparisons of the means have been done by unpaired Student´s t-test and ANOVA was used after treatment with Bonferroni´s correction for multiple testing when appropriate. Difference was considered significant if the p-value was below 0.05.

## RESULTS

### Effect of GP extract on PG in GK rats

The three-day treatment of GK rats with 800 mg/kg GP extract, or placebo extract, given orally twice a day had no significant effect on PG levels (from 9.9 ± 1.2 to 8.9 ± 0.6 mmol/L in GP treated, and from 8.5 ± 0.4 to 8.6 ± 0.3 mmol/L in placebo treated rats). In the three-week treatment, the PG concentrations were reduced significantly in GP treated rats from 9.8 ± 0.6 to 6.8 ± 0.4 mmol/L (*p*=0.027), while in the placebo rats, PG levels were slightly but not significantly decreased from 8.8 ± 0.8 to 7.5 ± 0.3 mmol/L.

### Effect of GP extract on IPGTT, SCITT and IPPTT in GK rats

**Figure 1:**
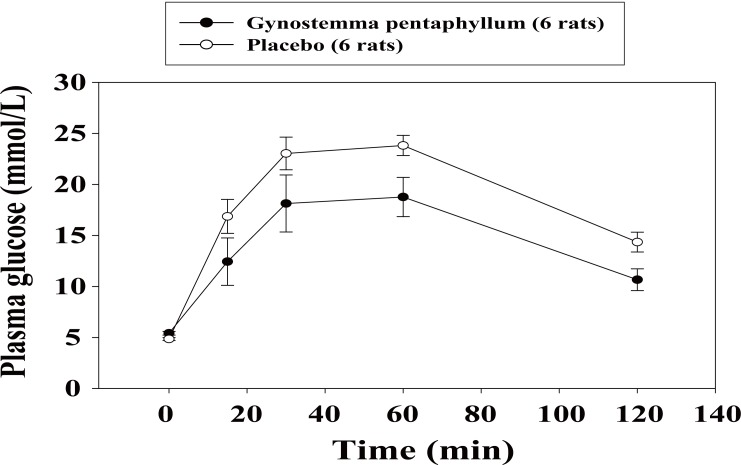
Mean of plasma glucose levels in the intraperitoneal glucose tolerance test after three-week treatment.

**Figure 2:**
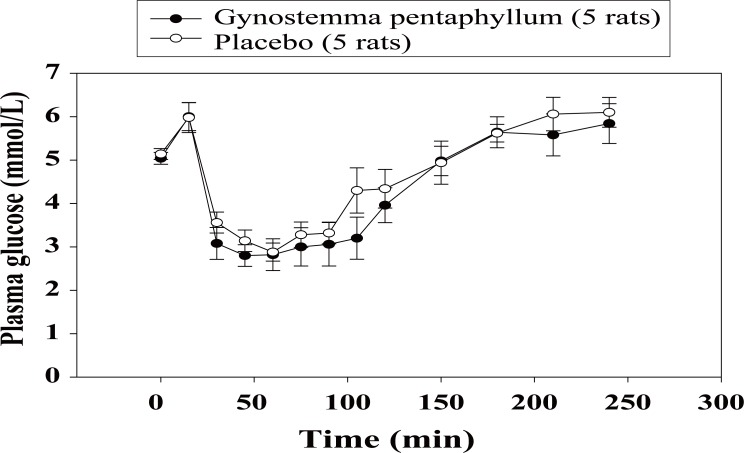
Mean of plasma glucose levels in the subcutaneous insulin tolerance test after three-week treatment.

**Figure 3:**
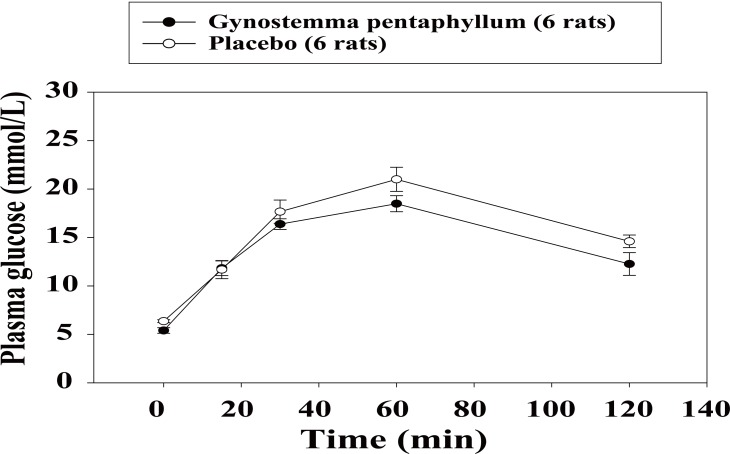
Mean of plasma glucose levels in intraperitoneal pyruvate tolerance test after three-week treatment.

In the IPGTT, the baseline glucose tolerance test (day 0) was similar in both groups, the areas under the glucose curves (AUCs) during 120 min (0-120 min) being 1995.1 ± 102.2 *vs.* 2029.5 ± 135.1 mmol/L, (*p*=0.843). However, after three-week treatment with GP extract, the glucose tolerance was significantly improved as compared to that in the placebo group with AUCs 1149.6 ± 200.0 *vs.* 1727.9 ± 95.5 mmol/L, respectively (*p*<0.05; Fig. 1). After three-week treatment, the PG concentrations in rats administered with s.c insulin (0.5 U/kg, SCITT) were reduced similarly in both groups, AUCs (0-240 min) (-149.7 ± 71.8 *vs.* -110.4 ± 65.1 mmol/L, *p*=0.696; Fig. 2). In the IPPTT, there were no significant differences in the total glucose responses between the two groups, AUCs (0-120 min) (1137.7±67.8 *vs.* 1240.1 ± 103.6 mmol/L; *p*=0.432; Fig. 3). However, when analysing the glucose response from minute 15 to minute 120, the AUC (15-120 min) was significantly lower in the GP group (415.5 ± 68.0 *vs.* 641.5 ± 41.8 mmol/L; *p*<0.05).

### Effect of GP extract on HGO levels in GK rats

**Figure 4:**
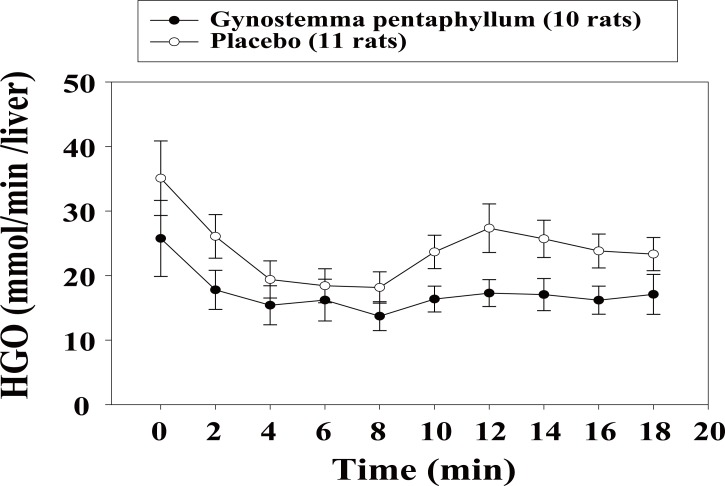
Mean of hepatic glucose output (HGO) after three-week treatment, (basal perfusion 0-8 min, and addition of 50 nM adrenaline 8-18 min).

After the three-day treatment with the GP extract, basal HGO was 20% lower in comparison with that in the placebo group (28.8 ± 6.5 *vs.* 36.1 ± 11.6 μmol/min). After three weeks, the basal HGO in GP treated rats was 27% lower (25.7 ± 5.9 *vs.* 35.1 ± 5.8 μmol/min in treated and placebo groups, respectively), but these differences were not statistically significant. However, in the three-week treated GK rats, AUCs for HGO during 18 minutes (0-18 min) was significantly decreased as compared with the placebo rats (302.8 ± 36.5 *vs.* 423.5 ± 44.7 μmol/min, *p*=0.05; Fig. 4). In addition, infusion of 50 nmol/L adrenaline beginning after 8 min of perfusion (8-18 min adjusted to the 8 min of perfusion) did increase the HGO in all rats but the response to adrenaline tended to be lower in the treated rats compared to the placebo rats (27.5 ± 8.9 *vs.* 61.0 ± 17.5 μmol/min, *p*=0.114).

### Effect of GP extract on hepatic glycogen content and GS activity in liver of GK rats

The three-week treatment with GP extract significantly reduced the hepatic glycogen content as compared to placebo group (18.3 ± 4.6 *vs.* 35.6 ± 2.9 mg/g liver, respectively, *p*<0.007). The hepatic glycogen synthase activity did not differ in the group treated with the GP extract (40.5 ± 6.3 percent) compared to that in placebo group (35.9 ± 6.2 percent).

## DISCUSSION

Based on our previous results in type 2 diabetic patients, suggesting that GP treatment exerted a potent anti-hyperglycemic effect by improving hepatic insulin sensitivity (11), we decided to explore whether GP extract modulates hepatic glucose production in GK rats.

Oral administration of GP extract for three days did not change the PG levels, whereas the long-term three-week treatment revealed significant decrease in PG. In addition, the three-week treatment with GP extract significantly improved glucose tolerance and reduced HGO compared to the placebo rats. Since the results of an insulin tolerance test reflecting the insulin sensitivity mainly in muscle and other extra-hepatic tissues did not differ between the two groups, it seems unlikely that the primary effect of GP extract is exerted on the extra-hepatic tissues. Thus, these findings suggest that GP-induced improvement in glucose tolerance in GK rats is accounted for, at least partly, by decreased HGO. Moreover, although addition of adrenaline did increase the HGO in all rats, the adrenaline effect tended to be suppressed in the GP extract treated rats.

HGO plays a prominent role in glucose homeostasis. Insulin decreases HGO by activating glycogen synthesis and glycolysis, and by suppressing gluconeogenesis (22). Glycogen is the intracellular stored form of glucose, and its levels in various tissues, particularly in liver and skeletal muscle, reflect the insulin activity stimulating glycogen synthase and inhibiting glycogen phosphorylase (23). We have shown that the hepatic glycogen content after three weeks of GP extract treatment was significantly lower than that of placebo treatment. In parallel, hepatic glycogen synthase activity did not differ between GP treated and placebo rats. In the liver, insulin-dependent glucose regulatory function is controlled by a number of different mechanisms. Among these, phosphotyrosine phosphatase 1B (PTP1B) is known to negatively modulate insulin action on the hepatic glucose metabolism through tyrosine dephosphorylation of the insulin receptor and/or insulin receptor substrates (24). GP extract has been demonstrated to inhibit PTP1B and this action has been linked to enhanced insulin sensitivity and improved glucose tolerance (25, 26). Interestingly, a more recent study has shown that hepatic glycogen content was significantly reduced in PTP1B -/- transgenic mice as compared to the wild-type controls (27). Therefore, it can be speculated that GP extract improves hepatic insulin sensitivity to some extent through inhibiting PTP1B. In addition, it seems likely that the improvement in hepatic insulin sensitivity is partly accounted for by a reduction of gluconeogenesis, as shown by the decreased glucose response during the pyruvate tolerance test in the GP extract treated GK rats.

In conclusion, oral administration of GP extract during three weeks to GK rats exerted anti-diabetic effects by reducing plasma glucose levels and suppressing HGO levels significantly. The mechanism behind the improved hepatic insulin sensitivity may relate to suppression of gluconeogenesis and inhibition of PTP1B.
